# Plasma Trimethylamine N-Oxide Levels Are Associated with Poor Kidney Function in People with Type 2 Diabetes

**DOI:** 10.3390/nu15040812

**Published:** 2023-02-04

**Authors:** Nora A. Kalagi, Rohith N. Thota, Elizabeth Stojanovski, Khalid A. Alburikan, Manohar L. Garg

**Affiliations:** 1 Department of Clinical Pharmacy, College of Pharmacy, King Saud University, Riyadh 11362, Saudi Arabia; 2 School of Biomedical Sciences and Pharmacy, University of Newcastle, Callaghan, NSW 2308, Australia; 3 Department of Biomedical Sciences, Macquarie University, Sydney, NSW 2109, Australia; 4 School of Mathematical and Physical Science, University of Newcastle, Callaghan, NSW 2308, Australia

**Keywords:** trimethylamine N-oxide, type 2 diabetes, chronic kidney disease, eGFR

## Abstract

Previous studies have linked elevated plasma trimethylamine N-oxide (TMAO) levels to poor renal function. The relationship between TMAO and chronic kidney disease (CKD) in type 2 diabetes (T2D) is still unclear. We investigated the association between plasma TMAO levels and CKD in patients with T2D. A cross-sectional study of 133 patients with T2D with or without CKD has been conducted. Blood biomarkers of kidney function, diabetes, and inflammation were assessed in the study participants. Plasma TMAO levels were quantified using UPLC-MS/MS. People with T2D and CKD exhibited significantly higher plasma TMAO levels [10.16 (5.86–17.45) µmol/L] than those without CKD [4.69 (2.62–7.76) µmol/L] (*p* = 0.002). Participants in the highest quartile of TMAO levels (>8.38 µmol/L) presented relatively elevated serum creatinine levels and a higher number of people with CKD than those in the lower quartiles. TMAO levels were significantly correlated with kidney function biomarkers, including estimated glomerular filtration rate and urinary albumin to creatinine ratio. The association between TMAO and CKD was evident (*p* < 0.0001) and remained significant after adjusting for risk factors of kidney disease, including age, gender, body mass index, duration of diabetes, and smoking. These findings suggest the association between plasma TMAO and CKD in patients with T2D.

## 1. Introduction

Chronic kidney disease (CKD) has been recognised as a major public health issue, with a high morbidity and mortality burden worldwide [1]. Diabetes mellitus, hypertension, aging, and obesity have all been linked to the progression of CKD. Previous studies have reported that the global prevalence of CKD among patients with type 2 diabetes (T2D) is 42.3%, primarily identified at early stages [2]. Diabetic nephropathy accounts for the majority of CKD cases in diabetic patients [3], with albuminuria and a low glomerular filtration rate (GFR) being the key predictors of diabetic kidney disease [4]. Elevated cardiovascular risk has been associated with CKD and T2D, leading to microvascular degradation and disease progression [5,6,7].

Recently, the gut microbiota has been identified as playing a critical role in a number of chronic diseases [8]. TMAO is produced in the human body by the gut microbiota degradation of choline-containing compounds, L-carnitine, and betaine into trimethylamine (TMA), followed by oxidation by the flavin mono-oxygenase (FMO3) enzyme in the liver [9]. Trimethylamine N-oxide (TMAO) has been identified as a novel risk factor for cardiovascular diseases [9] and metabolic disorders, including T2D [10,11,12,13,14,15] and CKD [16]. However, the mechanism by which TMAO promotes its atherogenic effect in these diseases is not entirely understood. TMAO cannot be metabolised in the human body and is eliminated primarily through the kidneys via urine [17]. Therefore, patients with impaired renal excretion rate are at a high risk of accumulating TMAO in the circulation [16].

Cross-sectional, case-control, and longitudinal studies have consistently demonstrated the significant contribution of high blood TMAO levels to CKD progression [16,18,19,20,21,22]. A strong inverse correlation has also been observed between TMAO and GFR [14,16,18,19,20,23,24,25,26,27,28,29]. Impaired renal function has drastically affected TMAO levels in circulation and was associated with increased mortality risk [16]. Furthermore, TMAO levels have been found to be elevated in patients with end-stage renal disease and patients on haemodialysis compared to individuals with normal kidney function [19,20,30]. On the other hand, animal studies have reported that long-term exposure to elevated TMAO levels has contributed to collagen deposition and progressive tubulointerstitial fibrosis [16]. Systemic inflammation and inflammatory cytokines such as C-reactive protein (CPR) have also been correlated with TMAO in CKD patients [20,29]. Whether TMAO could be used as a biomarker for evaluating renal function remains unknown.

Diabetic patients are more likely to develop microvascular complications such as nephropathy, which can worsen blood and urinary biomarkers of kidney function [31]. Most studies published to date on the relationship between TMAO and CKD were focused on individuals with established CKD, either mild to moderate or end-stage, or on dialysis compared to healthy individuals. However, only limited studies investigated this association in patients with T2D [29,32]. This study examined the association between plasma TMAO levels and CKD in people with T2D.

## 2. Materials and Methods

### 2.1. Study Design and Population

People with T2D (*n* = 133) were recruited in a prospective case-control study as previously described [33]. The study was approved by the University of Newcastle Human Research Ethics Committee (H-2018-0138) and the King Saud University Institutional Review Board (E-18-3073). All procedures were performed in accordance with the Declaration of Helsinki. Patients with acute illnesses or infections, currently on any antimicrobials or probiotics within three months of enrolment, and on haemodialysis or peritoneal dialysis were excluded.

Out of *n* = 133 participants with T2D, *n* = 12 (9%) had clinically confirmed diagnosis of CKD based on the criteria of estimated glomerular filtration rate (eGFR) less than 60 mL/min/1.73 m^2^ and/or the presence of albuminuria (urinary albumin to creatinine ratio (UACR) >300 mg/gm). This study was adequately powered to detect a significant difference in TMAO levels among those with T2D, with or without CKD. After providing informed consent, blood samples and clinical data were obtained from all patients. Patient interviews and medical records were used to assess demographic information and comorbidities.

### 2.2. Markers of Diabetes, Renal Function, and Systemic Inflammation

Fasting plasma glucose (FPG) and glycosylated haemoglobin (HbA1c) levels were measured in all patients with T2D. Blood and urinary markers of kidney function were also measured, including serum creatinine, blood urea nitrogen (BUN), and electrolytes such as sodium (Na), potassium (K), calcium (Ca), phosphorus, urine creatinine, and UACR. The eGFR was determined using the Chronic Kidney Disease Epidemiology Collaboration (CKD-EPI) formula [34,35]. Other markers associated with CKD include haemoglobin, albumin, alkaline phosphatase (ALP), total bilirubin, gamma-glutamyl transferase (GGT), and inflammatory markers such as high sensitivity C-reactive protein (hs-CRP) have also been assessed. All biochemical parameters were analysed according to certified standard protocols at King Saud University Medical City (KSUMC) central laboratory using the Dimension Vista^®^ 1500 Intelligent Lab System- version 3.10.2, DV311404 (Siemens Healthcare Diagnostics Inc., Tarrytown, NY, USA).

### 2.3. Quantification of Trimethylamine N-Oxide (TMAO) Levels in Plasma

Plasma TMAO levels were quantified using stable isotope dilution ultra-high-performance liquid chromatography with electrospray ionisation tandem mass spectrometry (UPLC/MS/MS) by (Waters Corporation, Milford, MA, USA) with d9-(trimethyl)-labelled as internal standard, and Acquity UPLC BEH HILIC column [100 mm × 2.1 mm, 1.7 µm particle size] (Waters Corporation, Milford, MA, USA), as previously described [33,36]. The mobile gradient phase was composed of acetonitrile and 15 mmol/L ammonium formate (pH 3.5) at a flow rate of 0.4 mL/min. The multiple reaction monitoring (MRM) was at m/z 76.1→58.2 for TMAO and 85.04→66.04 for the internal standard d9- TMAO.

### 2.4. Statistical Analysis

All data analyses were conducted using SPSS (version 27, SPSS Inc., Chicago, IL, USA) and graphs were created using GraphPad Prism (version 9). Participants’ characteristics were summarised according to T2D cases with or without CKD. Laboratory parameters and biomarker values were described using percentages for categorical variables, mean ± standard deviation (SD) or medians (IQR), and 25th to 75th quartiles range for continuous variables. For normally distributed data, the unpaired t-test and one-way ANOVA test evaluated the differences between study groups. Nonparametric tests were used for the measurements of non-normally distrusted data. The Mann–Whitney U test was used to compare data between the two patient groups, while the Kruskal–Wallis test compared patient data between plasma TMAO quartiles. Comparisons between categorical variables were analyzed by Pearson’s chi-squared test or Fisher’s exact test. Spearman’s rank correlation coefficient was applied to assess the association between TMAO and biomarkers of kidney disease. Multiple linear regression analysis was performed to examine the association between TMAO and CKD, adjusting for demographic variables (e.g., age, gender, BMI, duration of T2D, and smoking) as well as biomarkers of kidney function (e.g., UACR and eGFR) using a backward stepwise approach. The variables included in Model 2 were incorporated in the nomogram to predict CKD risk using “rms” package in R software version 4.2.2. A significance level of 0.05 was used for all analyses.

## 3. Results

### 3.1. Subject Characteristics and and Metabolic Parameters

Characteristics and metabolic parameters of study participants (T2D with CKD, *n* = 12, and without CKD, *n* = 121) are displayed in Table 1. Subjects with T2D and CKD were older compared to those without CKD (63 vs. 55 years, *p* = 0.02), had T2D for a median of 20 years, were more likely to be smokers (25% vs. 7.4%, *p* = 0.043), and were more likely to have macroalbuminuria (33.3% vs. 4.13%, *p* = 0.004). No significant differences were observed between study participants with and without CKD in terms of BMI, gender, FPG, ALP, calcium, phosphorus, total bilirubin, UACR, urine creatinine, and hs-CRP. HbA1c, haemoglobin, serum creatinine, BUN, eGFR, albumin, K, Na, and GGT levels were significantly higher in CKD participants than in those without CKD.

### 3.2. Plasma TMAO Levels and Markers of Kidney Disease

**Figure 1:**
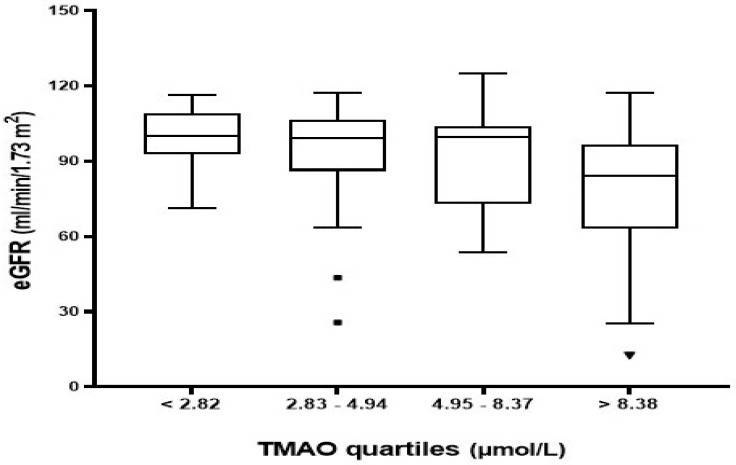
Estimated GFR distribution across plasma TMAO quartiles. Q1 (<2.82), Q2 (2.83–4.94), Q3 (4.95–8.37), and Q4 (>8.38).

Patients with CKD exhibited significantly higher plasma TMAO levels [10.16 (5.86–17.45) µmol/L] than patients without CKD [4.69 (2.62–7.76) µmol/L] (*p* = 0.002) as presented in Table 1. The total study population was stratified into quartiles based on plasma TMAO distribution to assess the changes in characteristics and laboratory markers associated with TMAO levels, as indicated in Table 2. TMAO levels were less than 2.82 µmol/L in the lowest quartile (Q1) and greater than 8.38 µmol/L in the highest quartile (Q4). Notably, patients in the highest quartiles of TMAO levels had a greater proportion of CKD, higher levels of serum creatinine and BUN, and lower levels of eGFR (Table 2 and Figure 1). Other variables such as gender, BMI, laboratory markers (e.g., kidney function tests and electrolytes), and hs-CRP were not statistically significantly different across TMAO quartiles.

#### 3.2.1. Correlation between Plasma TMAO Levels and Biomarkers of Kidney Disease

**Figure 2:**
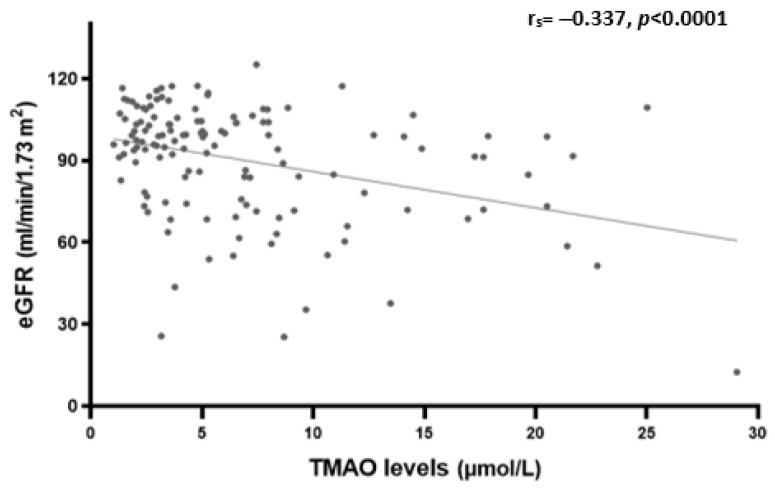
Spearman’s rank correlation analysis between plasma TMAO levels and eGFR.

Plasma TMAO levels were positively correlated with age (r_s_ = 0.198, *p* = 0.023), serum creatinine (r_s_ = 0.266, *p* = 0.002), BUN (r_s_ = 0.327, *p* < 0.0001), and UACR (r_s_ = 0.183, *p* = 0.046) (Table 3). However, a significant inverse correlation was observed with eGFR (r_s_ = −0.337, *p* < 0.0001) as shown in Figure 2.

#### 3.2.2. Association between Plasma TMAO Levels and CKD

Plasma TMAO levels were significantly associated with CKD in the non-adjusted Model 1 (β = 0.015, 95% CI [0.007, 0.024], *p* < 0.0001) as well as in Model 2 which adjusted for risk factors of CKD, including age, gender, BMI, duration of diabetes and smoking (β = 0.014, 95% CI [0.005, 0.022], *p* = 0.001) (Table 4). However, when the association was further adjusted for biomarkers of kidney disease including UACR and eGFR in models 3 and 4, respectively, plasma TMAO levels were no longer significantly associated with CKD as in Model 3 (β = 0.008, 95% CI [0.000, 0.016], *p* = 0.063) and Model 4 (β = 0.001, 95% CI [−0.005, 0.008], *p* = 0.706)].

#### 3.2.3. CKD Risk Prediction Nomogram

**Figure 3:**
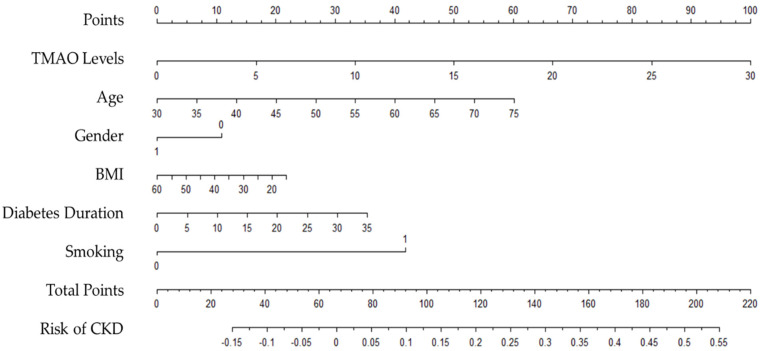
Nomogram to predict the risk of CKD in people with type 2 diabetes.

A nomogram was created to optimise the statistical predictive models into a single numerical probability estimate of CKD in the form of a graph. The nomogram was based on five parameters that were significant in multivariable analysis (Model 2) to predict the risk of CKD in T2D patients, including age, gender, BMI, diabetes duration, and smoking. The total point, which ranges from zero to two hundred and twenty, was computed by summing the points from each variable to determine CKD probability. The risk of CKD by total points was shown in the nomogram. The risk of CKD was lower than 10% for those below 90 points, and higher than 50% for those with over 200 points. The nomogram is shown in Figure 3.

Patients with more than 100 total points, for example, may have a 15% to 50% probability of developing CKD: TMAO levels greater than 7 µmol/L, age over 40 years, female gender, morbid obesity, BMI greater than 35 kg/m^2^, diabetes duration of more than 20 years, and smokers.

## 4. Discussion

This study explored the association between plasma TMAO levels and CKD in patients with T2D. Patients with CKD demonstrated higher levels of plasma TMAO than non-CKD patients. Plasma TMAO levels were positively correlated with biomarkers of renal function, including serum creatinine, BUN, and UACR, and were inversely correlated with eGFR. No significant relationship was found between plasma TMAO and other markers, including urine creatinine, serum electrolytes, ALP, total bilirubin, GGT, and hs-CRP. Elevated plasma TMAO levels were associated with CKD in the unadjusted model as well as when risk factors of CKD were adjusted for. However, the significant association was lost after further adjustment was made for CKD biomarkers, including UACR and eGFR. A simple monogram, based on the five variables that were significant in the multivariable analysis in Model 2 including TMAO level, age, BMI, gender, diabetes duration, and smoking demonstrated that TMAO levels contribute to the identification of T2D patients at high risk of developing CKD.

TMAO has been shown to aggravate kidney function decline and tubular interstitial injury, activate the inflammatory pathway via increasing p38 phosphorylation and human antigen R (HuR) level, upregulate NADPH oxidase 4 (NOX4), promote oxidative stress and nod-like receptor family pyrin domain containing three (NLRP3) inflammasome activation [37]. It has been speculated that TMAO can promote renal macrophage recruitment to induce tubular epithelial cell injury via the enhanced release of inflammatory cytokines [37]. CKD is also known to cause an imbalance in the gut microflora leading to a reduction in probiotics and a concurrent increase in toxigenic flora [38]; their toxic products may enter the host circulatory system and lead to sustained systemic inflammation.

Uncontrolled hyperglycaemia, particularly diabetes mellitus, remains a critical risk factor for CKD [39]. We [33] and others [13] have recently shown that TMAO levels are elevated in people with diabetes mellitus. In this study, we provide evidence that TMAO levels are further aggravated in those diagnosed with CKD. A recent study by Winther et al. showed that TMAO levels are elevated in individuals with T2D and albuminuria, placing them at a high risk of developing renal and cardiovascular disease [31], while the study by Al-Obaide et al. demonstrated higher TMAO levels in patients with T2D and advanced CKD [29]. In the current study, we report that TMAO levels are much higher in people with T2D who have progressed to develop CKD. Whether further increase in TMAO levels is the cause or consequence of progressing from T2D to CKD patients remains unknown. Higher plasma TMAO levels have been associated with an increased abundance of TMAO-producing bacteria in the intestinal microbiota of T2D patients with CKD [29]. In addition, TMAO is almost exclusively excreted by the kidneys [17], therefore, circulating levels can be expected to build up in CKD patients or those with renal impairment.

Accumulating evidence has demonstrated a significant association between TMAO and CKD [16,19,20,21,22], regardless of the patient’s diabetic status. However, the exact mechanism underlying the potential relationship between TMAO and CKD is still unclear. A linear incremental relationship has been noted between plasma TMAO levels and biomarkers of kidney histopathologic and functional impairment [16]. TMAO pathway has been found to contribute to kidney disease progression by dietary exposure to a choline-rich diet or TMAO directly, resulting in the development of tubule-interstitial fibrosis and dysfunction [16]. The study findings of an inverse correlation between TMAO and GFR substantiate previous findings suggesting the elimination of TMAO from the circulation to be mostly dependent on urinary excretion [22,40].

The study observations are in line with several previous studies in patients with cardiovascular diseases [23,24,25,26,27] and CKD [16,19,20,28]. Pelletier et al., on the other hand, observed only a modest correlation between TMAO and eGFR, which could be attributed to the study use of a different GFR formula than eGFR [22]. TMAO levels were also found to be positively correlated with serum creatinine and UACR as an early indicator of vascular injury in T2D, supporting earlier study findings [22,24,28,30]. In contrast to previous observations [20,30], this study found a nonsignificant correlation between TMAO levels and hs-CRP. The exact reason for the discrepancies in the findings between these studies and ours is unknown; however, these could be attributed to the study participants’ active disease and inflammatory state that may impact the outcome. TMAO metabolism is affected by the degree of CKD and haemodialysis status, as reported by other studies [20,30].

The association between plasma TMAO and CKD has been confirmed in the unadjusted model as well as after adjusting for CKD risk factors, including age, gender, BMI, diabetes duration, and smoking. However, when the model further adjusted for biomarkers of kidney disease such as UACR and eGFR, the association between TMAO and CKD was attenuated. This finding proposed a potential interaction between TMAO and biomarkers of kidney disease, suggesting TMAO as a surrogate marker for GFR and urine albumin as a predictor of poor outcomes in CKD patients [16]. Furthermore, our nomogram demonstrated that TMAO levels are one of the significant predictors of CKD progression in T2D patients. Increased TMAO levels have been shown to increase the likelihood of CKD development by up to 55% when combined with other T2D risk factors. This finding further supports the proposed link between TMAO and CKD in people with T2D.

The present study is the first in the Middle East to investigate the association between TMAO and blood markers of kidney disease in patients with T2D. We were able to understand the renal implications of TMAO and its correlation with poor renal outcomes in T2D patients. In this cross-sectional study, we have included only patients with a clinically confirmed diagnosis of T2D to control for any potential confounding produced by differences in diabetes diagnosis. The observational nature of the present study prohibits any inferences on the causality of TMAO and CKD in T2D patients. Patients from only one medical centre in the capital city of Saudi Arabia were included, limiting the generalizability of the findings. Further studies with a larger number of patients are warranted to verify the association between circulating TMAO levels and CKD with T2D.

In conclusion, our study has confirmed the association between circulating TMAO levels and CKD in a Saudi Arabian population where the dietary habits are different from the countries where the association has been previously established. Plasma TMAO levels are significantly associated with CKD, aggravated further in people with T2D, and correlated with biomarkers of renal function such as serum creatinine, eGFR, BUN, and UACR. Longitudinal and randomised controlled studies are warranted to establish if the progressive increase in TMAO and CKD is the cause or consequence of renal impairment in people with T2D. Identification of dietary and other lifestyle factors that can influence circulating TMAO levels may further enhance investigations into the prevention of T2D and CKD.

## Figures and Tables

**Table 1:** Participant characteristics and metabolic parameters of patients with T2D, with or without CKD.

Variables	Type 2 Diabetes		
	without CKD (*n* = 121)	with CKD (*n* = 12)	*p*-Value *
**Demographics**			
Age (years) ^a^	55 ± 9	63 ± 8	0.020
Gender, No. (%)			0.262
Male	23 (19)	4 (33)	
Female	98 (81)	8 (66.7)	
BMI (kg/m^2^) ^b^	30.96 (28.31–34.81)	31.52 (26.91–36.52)	0.956
Smoking (yes), No. (%)	9 (7.4)	3 (25)	0.043
**Medical History**			
Diabetes duration (years) ^b^	11 (6–20)	20 (15–20)	0.007
**Laboratory Parameters**			
FPG, mmol/L ^b^	7.82 (8.00–10.00)	9.25 (8.16–12.58)	0.051
HbA_1C_, % ^b^	8.2 (7.2–9.4)	9.3 (8.7–10.1)	0.036
Haemoglobin, gm/L ^a^	132 ± 13	122 ± 17	0.021
Serum creatinine, µmol/L ^b^	60 (51–73)	128 (94–168)	<0.0001
BUN, mmol/L ^b^	4.20 (3.30–5.30)	9.75 (6.35–13.65)	<0.0001
GFR, mL/min/1.73 m^2 b^	98.75 (84.83–105.59)	47.49 (30.51–55.22)	<0.0001
Albumin, gm/L ^a^	36.86 ± 2.98	34.77 ± 3.45	0.024
Alkaline phosphatase, unit/L ^b^	89 (64–108)	97 (74–143)	0.212
K, mmol/L ^a^	4.49 ± 0.43	4.82 ± 0.50	0.015
Na, mmol/L ^b^	140 (138–141)	137 (135–140)	0.005
Ca^+^, mmol/L ^b^	2.34 (2.25–2.42)	2.36 (2.30–2.42)	0.574
Phosphorous, mmol/L ^b^	1.24 (1.11–1.36)	1.38 (1.20–1.48)	0.073
GGT, unit/L ^b^	24 (18–35)	52 (36–96)	0.001
Total Bilirubin, µmol ^b^	6.55 (5.48–8.41)	6.61 (5.68–10.82)	0.572
UACR, mg/gm ^b^	12.80 (6.68–32.11)	154.07 (5.10–446)	0.064
Microalbuminuria, No. (%)	23 (19)	3 (25)	0.702
Macroalbuminuria, No. (%)	5 (4.13)	4 (33.3)	0.004
Urine creatinine, µmol/L ^b^	7149 (3885–9509)	3637 (2836–7170)	0.069
Hs-CRP, mg/L ^b^	3.9 (1.3–8.3)	1.6 (1.2–7.7)	0.591
TMAO, µmol/L ^b^	4.69 (2.62–7.76)	10.16 (5.86–17.45)	0.002

CKD, chronic kidney disease; BMI: body mass index; FPG, fasting plasma glucose; HbA1c, glycosylated haemoglobin; GFR, glomerular filtration rate; GGT, gamma-glutamyl transferase, BUN, blood urea nitrogen; K, potassium; Na, sodium; Ca, calcium; UACR, urinary albumin to creatinine ratio; Hs-CRP, high sensitivity C-reactive protein; TMAO, trimethylamine N-oxide. ^a^ Data are presented as mean ± standard deviation). The two-sample t-test was used for the comparison. ^b^ Data are presented as median (IQR) (25th–75th percentiles). Mann–Whitney U test was used in the comparison. * *p* < 0.05, statistically significant difference; *p* > 0.05, no statistically significant difference.

**Table 2:** Participant characteristics by quartiles of TMAO levels.

		Quartiles of TMAO				
	*n*	Q1 (*n* = 32)	Q2 (*n* = 34)	Q3 (*n* = 34)	Q4 (*n* = 33)	*p*-Value ^c^
TMAO, µmol/L		< 2.82	2.83–4.94	4.95–8.37	> 8.38	
**Demographics**						
Age (years) ^a^	133	54 ± 8	53 ± 10	55 ± 10	60 ± 8 ^2^	0.011
Gender, No. (%)						0.531
Male	27	5 (15.6)	9 (26.5)	5 (14.7)	8 (24.2)	
Female	106	27 (84.4)	25 (73.5)	29 (85.3)	25 (75.8)	
BMI (kg/m^2^) ^b^	133	30.8 (28.58–33.17)	31.24 (28.21–36.45)	31.80 (29.50–37.53)	30.78 (27.48–34.00)	0.425
Smoking (yes), No. (%)	12	3 (9.4)	4 (11.7)	2 (5.9)	3 (9.1)	0.867
**Medical History**						
Kidney disease (yes), No. %	12	0	2 (5.9)	2 (5.9)	8 (24.2) ^1,2,3^	0.004
Diabetes duration	133	15 (8–21)	10 (8–15)	11 (5–20)	15 (8–20)	0.437
**Laboratory Parameters**	133					
FPG, mmol/L ^b^		8.25 (7.11–10.86)	7.4 (6.25–10.04)	8.24 (5.50–10.30)	7.2 (5.31–9.35)	0.488
HbA_1C_, % ^b^		8.5 (7.85–9.45)	8.2 (7.20–9.60)	8.6 (7.20–10)	8.2 (7.20–9.30)	0.770
Haemoglobin, gm/L ^a^		134 ± 12	132 ± 14	130 ± 15	127 ± 14	0.139
Serum creatinine, µmol/L ^b^		54 (50–69)	63 (54–80)	61 (49–82)	71 (60–90) ^1^	0.016
BUN, mmol/L ^b^		3.6 (3.2–4.4)	4.7 (3.4–5.4)	4.5 (3.7–5.7)	5.6 (3.7–7.2) ^1^	0.002
GFR, mL/min/1.73 m^2 b^		99.96 (93.02–108.94)	99.10 (86.15–105.86)	99.48 (73.72–104.06)	84.10 (65.94–94.35) ^1,2^	0.001
Albumin, gm/L ^a^		36.44 ± 2.61	37.41 ± 3.34	36.45 ± 3.16	36.36 ± 3.13	0.455
Alkaline phosphatase, unit/L ^b^		89.5 (67.50–114)	81.5 (59–108)	93 (67–108)	94 (76–113)	0.424
K, mmol/L ^a^		4.4 ± 0.5	4.5 ± 0.4	4.6 ± 0.4	4.6 ± 0.5	0.196
Na, mmol/L ^b^		139 (138–141)	140 (138–141)	140 (138–142)	140 (138–141)	0.780
Ca^+^, mmol/L ^b^		2.39 (2.30–2.43)	2.34 (2.25–2.39)	2.35 (2.28–2.42)	2.34 (2.35–2.39)	0.558
Phosphorous, mmol/L ^b^		1.25 (1.10–1.37)	1.23 (1.09–1.35)	1.27 (1.12–1.38)	1.27 (1.16–1.48)	0.443
Total Bilirubin, µmol ^b^		6.87 (6.44–8.70)	6.30 (4.82–8.32)	6.61 (5.26–8.41)	6.55 (5.55–9.67)	0.228
UACR, mg/gm ^b^		9.12 (5.91–32.95)	11.28 (5.90–17.43)	15.47 (8.13–78.25)	22.25 (7.60–159.86)	0.154
Urine creatinine, µmol/L ^b^		7403 (3979–10475)	6832 (3637–8506)	6622 (4569–9843)	4638 (2648–9202)	0.614
Hs-CRP, mg/L ^b^		3.6 (2.4–7.8)	2.2 (0.9–8.8)	4.1 (1.1–6.0)	2.9 (1.2–7.2)	0.399

Trimethylamine N-oxide; BMI: body mass index; FPG, fasting plasma glucose; HbA1c, glycosylated haemoglobin; GFR, glomerular filtration rate; GGT, gamma-glutamyl transferase, BUN, blood urea nitrogen; K, potassium; Na, sodium; Ca, calcium; UACR, urinary albumin to creatinine ratio; Hs-CRP, high sensitivity C-reactive protein. ^a^ Data are presented as mean ± standard deviation. One-way ANOVA was used in the comparison. ^b^ Data are presented as median (IQR) (25th–75th percentiles). Kruskal–Wallis test was used in the comparison. ^c^ *p* < 0.05, Statistically significant difference; *p* > 0.05, no statistically significant difference. ^1^ Q4 is significantly different than Q1 with *p* < 0.05; ^2^ Q4 is significantly different than Q2 with *p* < 0.05; ^3^ Q4 is significantly different than Q3 with *p* < 0.05.

**Table 3:** Spearman’s rank correlation coefficient to assess the relationships between TMAO and markers of kidney function.

Variables	r_s_	*p*-Value *
Age	0.198	0.023
BMI	−0.037	0.672
Diabetes duration	0.018	0.841
Serum creatinine	0.266	0.002
BUN	0.324	<0.0001
Albumin	−0.043	0.623
Alkaline phosphatase	0.079	0.367
Total Bilirubin	−0.048	0.581
K	0.122	0.161
Na	0.081	0.356
Ca	−0.007	0.376
Phosphorous	0.113	0.196
GGT	−0.107	0.220
UACR	0.183	0.046
Urine creatinine	−0.088	0.342
Hs-CRP	−0.100	0.404

BMI: body mass index; BUN, blood urea nitrogen; K, potassium; Na, sodium; Ca, calcium; GGT, gamma-glutamyl transferase, UACR, urinary albumin to creatinine ratio; Hs-CRP, high sensitivity C-reactive protein. * *p* < 0.05, statistically significant correlation; *p* > 0.05, no statistically significant correlation.

**Table 4:** Association between plasma TMAO levels and CKD using multiple linear regression models.

Outcome—CKD	TMAO Levels, µmol/L		
	β	*p*-Value	95% CI
Model 1 (Unadjusted)	0.015	<0.0001 *	0.007, 0.024
Model 2	0.014	0.001 *	0.005, 0.022
Model 3	0.008	0.063	0.000, 0.016
Model 4	0.001	0.706	−0.005, 0.008

Model 1 is non-adjusted; Model 2 is adjusted for age, gender, BMI, diabetes duration, and smoking; Model 3 is adjusted for model 2 + UACR; Model 4 is adjusted for model 3 + eGFR. * Statistically significant association (*p* < 0.05).

## Data Availability

The datasets used and analyzed during the current study are available by contacting the corresponding author upon request.
